# Butterfly Clap–Fling Flight Mechanisms Observed by Schlieren Imaging for the Design of Bio-Inspired Micro Air Vehicles

**DOI:** 10.3390/biomimetics11030184

**Published:** 2026-03-04

**Authors:** Emilia-Georgiana Prisăcariu, Sergiu Strătilă, Oana Dumitrescu, Mihail Sima, Raluca Andreea Roșu, Iulian Vlăducă

**Affiliations:** Romanian Research and Development Institute for Gas Turbines, 220D Iuliu Maniu Blvd., 061126 Bucharest, Romania; sergiu.stratila@comoti.ro (S.S.); oana.dumitrescu@comoti.ro (O.D.); mihail.sima@comoti.ro (M.S.); raluca.rosu@comoti.ro (R.A.R.); iulian.vladuca@comoti.ro (I.V.)

**Keywords:** butterfly, low Reynolds regimes, schlieren visualization, wingtip vortex

## Abstract

This paper investigates the flight kinematics and unsteady aerodynamics of butterfly flight using high-speed schlieren imaging. Butterfly trajectories are reconstructed to examine flight control mechanisms, with particular emphasis on thorax-driven manoeuvring and body reorientation. By reconstructing free-flight trajectories utilizing image recognition algorithms, we isolate the mechanisms of flight control, with particular emphasis on how thoracic oscillation drives manoeuvring and body reorientation. Phase-resolved analysis reveals distinct wingbeat modes, including clap-and-fling motions associated with hovering and low-speed ascent. Schlieren visualization further captures a detailed view of the wake topology, displaying the formation and evolution of wingtip vortices during the downstroke, as well as attached and entrained flow structures during cupped wing configurations. The results demonstrate the strong coupling between body dynamics, wing kinematics, and wake structure, highlighting how butterflies combine aerodynamic and inertial mechanisms to achieve efficient lift generation and control. These findings provide biomimetic insights relevant to the design of flapping wing micro air vehicles, particularly for low-speed flight, hover efficiency, and passive stability and control through body–wing coupling.

## 1. Introduction

### 1.1. Background: Butterfly Flight and Unsteady Aerodynamics

Insects provide some of the most compelling natural examples of efficient flight at low Reynolds numbers, a regime in which conventional steady aerodynamic assumptions break down. Typical insect flight occurs at Reynolds numbers (Re) ranging from 10 to 10^4^ [1], where viscous effects, flow separation, and unsteady phenomena dominate aerodynamic force production [2]. In this regime, lift and thrust are generated primarily through time-dependent mechanisms rather than through the quasi-steady airfoil behaviour characteristic of higher-Reynolds-number flight [3,4]. The Reynolds number is critical because it determines the balance between inertial and viscous forces, thereby governing the unsteady vortex-dominated mechanisms that characterize butterfly flight and low-scale flapping wing vehicles.

Extensive experimental and theoretical studies have shown that insects exploit a range of unsteady aerodynamic strategies, including leading edge vortex stabilization [5,6,7], wake capture [8], rotational circulation [9], and clap-and-fling interactions. These mechanisms enable insects to generate lift coefficients well beyond those predicted by steady state theory, allowing sustained hovering, agile manoeuvring, and efficient flight at low forward speeds.

Table 1 highlights the wide range of Reynolds numbers encountered in insect flight, spanning nearly four orders of magnitude from miniature insects such as thrips to large flying species such as hawkmoths and dragonflies. This broad range reflects fundamental changes in flow physics, with viscous-dominated regimes at very low Reynolds numbers and increasingly inertial effects governing the aerodynamics of larger insects.

To fly in this viscous regime, insects employ a suite of unsteady manoeuvres, most notably the “clap-and-fling” mechanism first described by Weis-Fogh [15,16,17]. By bringing the wings together at the end of the upstroke (clap) and peeling them apart at the leading edge (fling), insects can rapidly establish circulation and generate a high-lift leading-edge vortex (LEV) immediately at the start of the stroke. This is often augmented by wake capture, where the wing intercepts the vorticity shed during the previous stroke to recover energy. However, these aerodynamic forces are not generated in isolation; they are linked to the insect’s body dynamics. Body–wing coupling—the interaction between the oscillating wings and the inertial movement of the thorax—plays a crucial role in flight control. The thorax does not merely serve as a rigid fuselage but actively oscillates in response to wing torque, contributing to passive stability and energy storage, a phenomenon that is critical for efficient flight control but remains difficult to model.

Despite the wealth of literature on insect aerodynamics, research has disproportionately focused on Diptera (flies) [18,19,20,21] and Odonata (dragonflies) [22,23,24,25]—species characterized by relatively high wingbeat frequencies, small stroke amplitudes, and rigid wings. Butterflies (Lepidoptera), by contrast, remain comparatively understudied despite their distinctive flight mechanisms. They possess unusually large, low-aspect-ratio wings that exhibit significant flexibility and aeroelastic deformation, operating at much lower frequencies than their dipteran counterparts [26]. Butterfly flight involves a downward wingbeat primarily generating lift, followed by an upward stroke that contributes to thrust production. Sunada et al. [27] showed that the initial wing-opening motion during takeoff produces the first lift peak, highlighting the importance of coordinated wing and body motions during transient flight phases. Zhu et al. [28] used a high-fidelity fluid–structure interaction model to study butterfly flight. It was found that a baseline forewing–hindwing configuration (Δα = 0°) with moderate flexibility (E = 4 GPa) maximizes lift, thrust, and propulsive efficiency. This effect is achieved through the formation of complex vortex structures, highlighting the critical role of wing morphology and flexibility.

In addition, butterflies are known for their erratic, bobbing flight trajectories and complex thoracic pitching motions, which make experimental stabilization and repeatability challenging. Recent studies further indicate that butterfly flight is characterized by low-frequency, large-amplitude flapping, coordinated lead–lag motion of the forewings during the downstroke, and substantial wing twist (feathering), all of which play a key role in their aerodynamic performance [29]. While this erratic flight style challenges traditional analyses, it represents a unique functional solution for flight at very low speeds and high manoeuvrability, requiring advanced flow visualization techniques to resolve the intricate flow structures involved.

### 1.2. Flow Visualization in Insect Flight

Experimental analysis of insect aerodynamics relies heavily on flow visualization techniques to elucidate the complex, unsteady flow structures generated by flapping wings. Traditional approaches, such as particle image velocimetry (PIV) and smoke visualization, have been widely used to quantify flow fields. PIV provides quantitative velocity field data by tracking seeded particles illuminated by a laser sheet. However, PIV presents significant challenges when working with live insects; the requisite seeding particles can affect the insect’s respiration or behaviour, and high-intensity lasers pose safety risks to the specimen’s eyesight and thermal regulation. Similarly, smoke wire or smoke rake visualization, while effective for observing pathlines, introduces foreign matter into the control volume that may induce coughing or erratic flight in biological specimens, or physically disturb the delicate boundary layers on small wings. These limitations are particularly pronounced in butterflies, whose large, flexible wings and sensitive flight behaviours require non-intrusive observation methods.

In contrast, optical methods based on the deflection of light—specifically shadowgraphs and schlieren photography—offer a distinct advantage. While shadowgraph detects the second derivative of the refractive index (showing flow boundaries), schlieren imaging is sensitive to the first derivative, making it highly effective for visualizing density and velocity gradients.

For the study of butterfly flight, schlieren photography stands out as the optimal choice due to its non-intrusive, seedless nature. By exploiting the refractive index changes caused by the insect’s metabolic heat or slight pressure variations, this technique allows for the visualization of the flow field without the need for physical seeding or potentially toxic tracers. This “seedless” approach is critical for capturing natural, unperturbed flight behaviours. Furthermore, schlieren is particularly adept at capturing the formation of leading-edge vortices (LEVs) and the entrainment of fluid, providing a high-contrast visualization of the thermal plumes and wake structures that define the aerodynamic footprint of the insect.

Building on non-intrusive visualization techniques, numerous studies have combined high-speed imaging, schlieren, and numerical simulations to investigate butterfly flight. Zhang et al. [26] have shown that forewing flapping, abdominal swing, and body pitch are strongly coupled, regulating three-dimensional vortex structures and the clap-and-peel mechanism, which directly affects force generation and flight performance. Fang et al. [30] demonstrated that body angle and chordwise wing deformation significantly influence lift, with decreased body angle and moderate wing flexibility enhancing forward speed and upstroke efficiency, while excessive deformation can alter force direction. Wu et al. [31] revealed that hovering lift arises mainly from attached leading-edge vortices on the forewings, whereas the early shedding of hindwing trailing-edge vortices contributes minimally (~10%) to weight support. Substantial body pitching (~90°) ensures vertical force generation and pitching balance, providing insights for bio-inspired flapping wing robots.

The combination of high-speed imaging, schlieren photography, and advanced numerical simulations has significantly advanced our understanding of butterfly flight. Schlieren and other flow visualization techniques allow the capture of unperturbed, seedless aerodynamic structures such as LEVs, TEVs, and wakes. When coupled with quantitative modelling, these methods reveal the intricate interactions between wing kinematics, body pitching, and vortex dynamics that govern force generation, flight performance, and stability.

### 1.3. Motivation and Objectives

Understanding flight mechanisms is not only fundamental to comparative biomechanics, but is also critical for the engineering of flapping wing micro air vehicles (FW-MAVs). As the demand for MAVs capable of hovering and manoeuvring in confined spaces grows, the highly manoeuvrable and energy-efficient flight of insects offers a rich template for biomimetic design, particularly regarding stability, gust rejection, and vertical ascent. Bio-inspired MAVs face challenges analogous to those encountered by insects: low-speed operation, limited actuation power, and strong sensitivity to environmental disturbances. Insects offer proven solutions to these challenges through their integration of flexible wings, body–wing coupling, and adaptive flight kinematics, making detailed studies of their flight invaluable for the development of robust, manoeuvrable, and efficient micro-scale aerial platforms.

Despite progress in bio-inspired aerodynamics, the complex flight mechanisms of butterflies remain under-explored compared with dipteran (fly) or hymenopteran (bee) species. Existing studies have largely relied on tethered specimens or numerical simulations, which may not fully capture the nuances of free-flight manoeuvring. This research addresses this gap by applying high-speed optical diagnostics to freely flying butterflies.

The primary motivation for this work is to investigate unsteady aerodynamic mechanisms, specifically the “clap-and-fling” interaction between the wings that enhances lift and thorax-driven manoeuvres that control flight path. Additional goals include visualizing plume-assisted ascent and the rapid dynamics of inversion and righting reflexes. Understanding these mechanisms is not merely of biological interest; it directly informs the design of FW-MAVs by revealing how butterflies manage stability and agility through morphological adaptations, such as wing cupping and wake capture, under unsteady conditions.

Accordingly, this study aims to design and implement a high-speed schlieren imaging system capable of resolving the fine-scale flow structures of free-flying butterflies without perturbing their natural behaviour. It seeks to visualize and characterize the flow features associated with the “clap-and-fling” mechanism and the resulting vortex shedding, while analysing the aerodynamic role of wing flexibility and cupping during the upstroke and their contribution to lift generation. Additionally, the research investigates the relationship between body kinematics, particularly thorax movement, and wake topology during rapid manoeuvring and stabilization.

To the authors’ knowledge, this represents the first high-speed schlieren visualization of butterfly flight, and the first direct observation of attached flow on a cupped butterfly wing during the upstroke.

## 2. Experimental Setup and Methods

### 2.1. Schlieren System and Imaging Parameters

**Figure 1:**
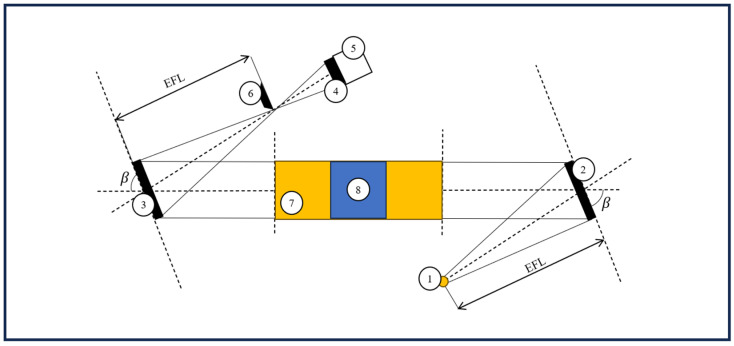
Schlieren Z-type configuration: 1—light source, 2—first parabolic mirror, 3—second parabolic mirror, 4—camera lens, 5—high-speed camera (CMOS), 6—knife edge, 7—test area, 8—transparent glass container. EFL is the effective focal length of the parabolic mirror and β is the mirror off-axis tilt angle.

**Figure 2:**
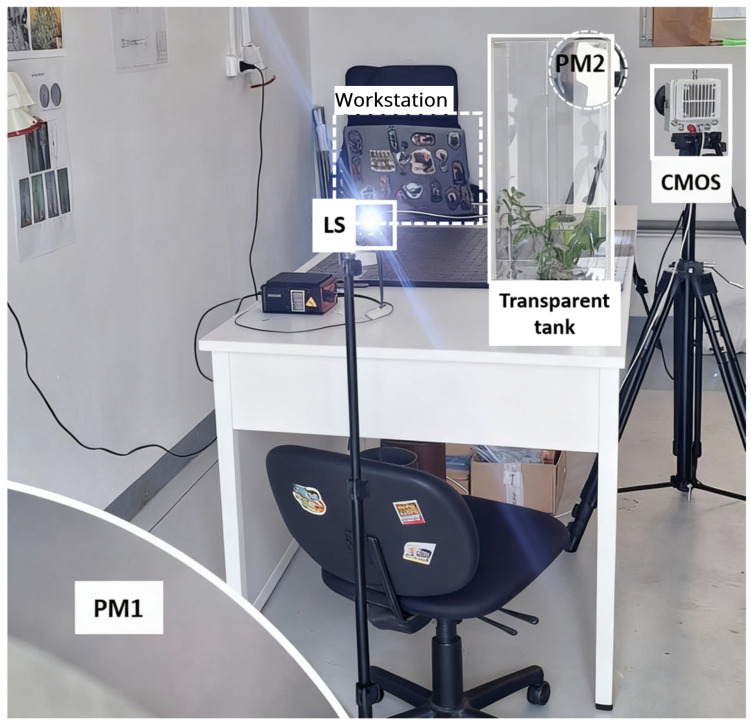
Physical setup of the Z-type schlieren system in the laboratory environment (where LS—light source, PM1 and PM2—parabolic mirrors).

**Figure 3:**
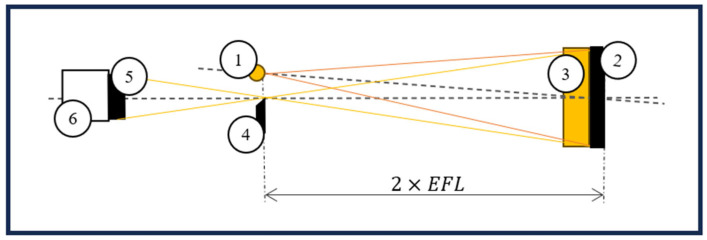
Schlieren single mirror configuration: 1—light source, 2—parabolic mirror, 3—test area, 4—knife edge, 5—camera lens, 6—high-speed camera (CMOS).

Two schlieren setups were used for capturing high-speed recordings of butterflies in free flight: a classical Z-type system (optical sketch presented in Figure 1 and physical setup presented in Figure 2) and a single-mirror configuration (sketch presented in Figure 3). The second configuration was used to provide better focus, due to the reduced size of the second airborne species analysed. The Z-type configuration was selected for the larger Iphiclides podalirius specimen, so as to allow confined flight within a transparent enclosure while preserving wide-field schlieren sensitivity. In contrast, a single-mirror configuration was used for the smaller Pieris rapae to enable free-flight observation with improved optical focus and reduced alignment complexity. The selection of each device was therefore guided by specimen size, required field of view, and the need to balance confinement control with natural flight behaviour.

These aspects are described in Section 2.2. Table 2 provides equipment specification used in both schlieren configurations.

### 2.2. Test Conditions and Specimen

**Figure 4:**
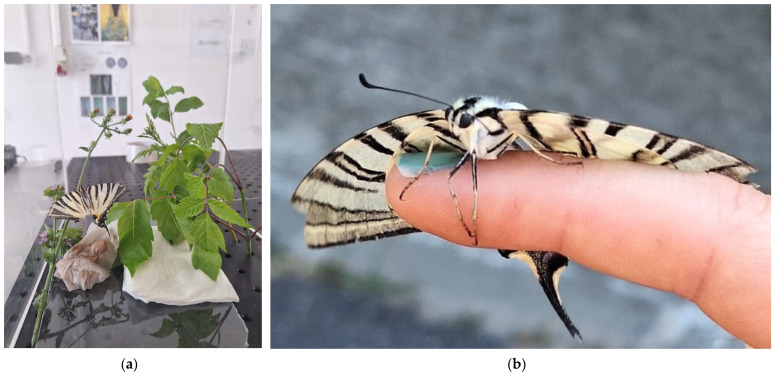
Iphiclides podalirius in (**a**) transparent tank (**b**) view from the front.

**Figure 5:**
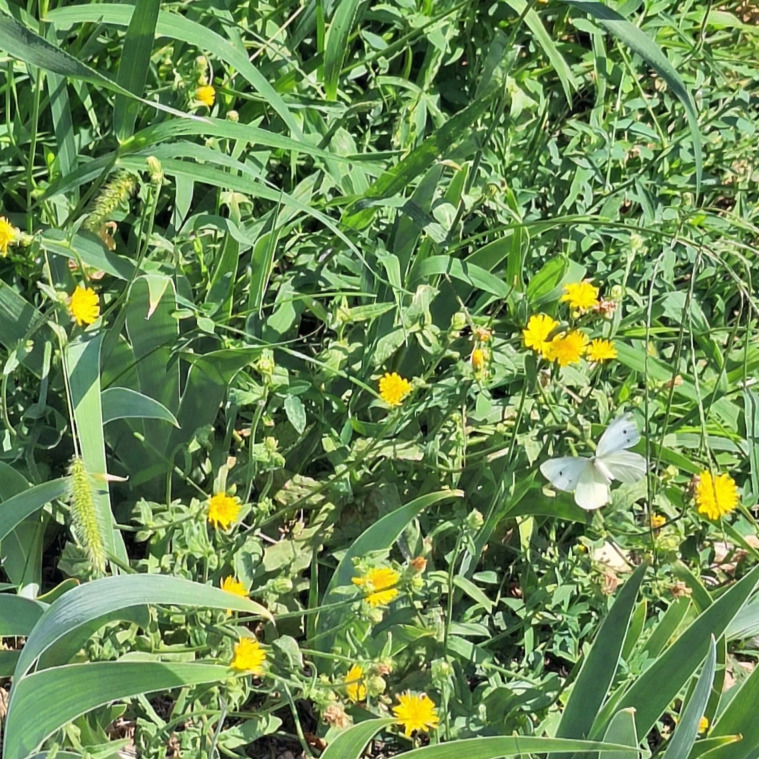
Pieris rapae (Small White) in COMOTI’s backyard pasture, before experimentation.

Experiments were conducted on two butterfly species with distinct morphological and flight characteristics: *Iphiclides podalirius* (Scarce Swallowtail) (Figure 4) and Pieris rapae (Small White) (Figure 5). These species were selected to provide representative cases of butterfly flight across different size scales and wing morphologies, while remaining accessible under laboratory conditions. The *Iphiclides podalirius* had a missing wing tail and several other wing defects, as it was captured from the wild.

Flight experiments were performed under ambient laboratory conditions without any imposed external flow or artificial seeding. For *Iphiclides podalirius*, flight was conducted inside a transparent enclosure positioned within a Z-type schlieren optical setup. The enclosure limited large-scale translational motion while preserving natural wing kinematics and allowing controlled visualization of the surrounding flow field. Natural convection within the enclosure was present and contributed to the background schlieren signal. Although the *Iphiclides podalirius* specimen exhibited minor wing damage, the recorded flow structures and kinematic patterns were consistent across cycles and were not dominated by asymmetrical deformation. As the study emphasizes qualitative vortex visualization and kinematic reconstruction rather than force quantification, the defect did not affect the primary aerodynamic observations.

In contrast, Pieris rapae was recorded in free flight within the laboratory environment using a single-mirror schlieren configuration. In this case, the butterfly was allowed to move freely in the measurement volume, with flow structures arising solely from the insect’s wing motion and ambient air convection.

Experiments were conducted under standard laboratory conditions (temperature 20–24 °C, relative humidity 40–60%, atmospheric pressure approximately 101 kPa).

The study involved one specimen of each species (Iphiclides podalirius and Pieris rapae), selected for controlled high-speed optical documentation of representative flight mechanisms rather than statistical population analysis.

The butterflies were kept for at most 20 min for video recording, after which they were put back in the pasture. The laboratory environment had air conditioning and shade, which are not ideal for butterflies. Rotten fruit and damped cotton were provided to ease the butterfly’s conditions between release phases.

In all experiments, no tracer particles, smoke, or imposed flow fields were introduced. The schlieren system therefore visualized only naturally occurring refractive index gradients generated by the butterflies’ motion and by background thermal convection, ensuring fully non-intrusive flow measurements.

### 2.3. Observations from Butterfly Schlieren Imaging

In classical wake analysis, the aerodynamic forces acting on a flapping body can be derived using wake deficit and impulse-based formulations, as proposed in [36]. The corresponding expressions are reported below for completeness, as they provide the theoretical framework commonly used to relate wake velocity deficits and vorticity distributions to aerodynamic force production. However, it should be emphasized that the present schlieren measurements visualize refractive index gradients rather than providing full velocity field reconstruction. Therefore, the equations are included as a reference framework for interpreting the observed vortex structures, rather than as directly applied quantitative force integrations in this study.

The net thrust $T_{net}$ is obtained from the classical wake deficit formulation, where ρ denotes the air density, $U_{\infty }^{\prime }$ represents the local free-stream velocity varying along the $y^{\prime }$-direction, and $u^{\prime }$ is the induced velocity component defined relative to the free-stream velocity. The free-stream direction is aligned with the x-axis.

The impulse-based formulation further expresses the aerodynamic force components in terms of wake vorticity. The impulse aligned with the flight direction is defined through the streamwise integration of the spanwise vorticity component. In these expressions, $\omega_{x},\omega_{y},\omega_{z}$ denote the vorticity components evaluated in the local plane, x and y represent spatial coordinates measured relative to the wake centre, Δz is the spacing between successive measurement planes, and n is the index of the horizontal measurement plane used in the discrete summation.

**Figure 6:**
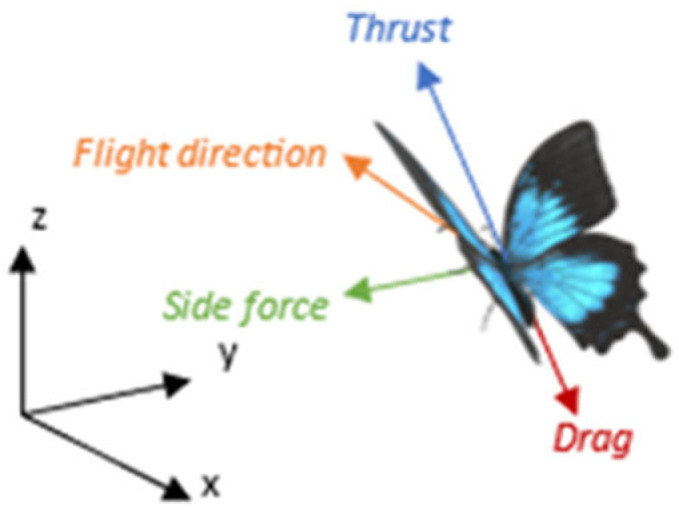
Forces acting on an airborne butterfly.

The lateral impulse $I_{\mathrm{sides}}$ and the vertical impulse $I_{z}$ are defined analogously through the corresponding moment integrals of the vorticity field. The representation of these force components acting on an airborne butterfly is illustrated in Figure 6.

(1) $$T_{\mathrm{net}}=\rho \iint_{\begin{array}{c} wake\;\\ area \end{array}}u^{\prime }\left(y^{\prime },z\right)\left(U_{\infty }^{\prime }\left(y^{\prime },z\right)+u^{\prime }\left(y^{\prime },z\right)\right)\;dy^{\prime }dz;$$

(2) $$I_{\mathrm{flight}\;\mathrm{direction}}=\rho \Delta z\sum_{n_{z}=1}^{N_{z}}\iint_{\begin{array}{c} wake\;\\ area \end{array}}\omega_{z}^{\prime }\left(x^{\prime },y^{\prime },n_{z}\right)s_{y^{\prime }}\left(x^{\prime },y^{\prime },n_{z}\right)\cdot dx^{\prime }dy^{\prime };$$

(3) $$I_{x}=\rho \sum_{n}\iint y\omega_{z}dxdy∆z\;I_{sides}=\rho \sum_{n}\iint x\omega_{z}dxdy∆z\;I_{z}=\rho \sum_{n}\iint \left(x\omega_{y}-y\omega_{x}\right)dxdy∆z$$

Because velocity-resolved wake data are not available from the current schlieren recordings, the force components described by Equations (1)–(3) are not evaluated numerically in Section 3. Instead, the equations serve to contextualize the qualitative vortex observations within an impulse-based aerodynamic interpretation.

Schlieren imaging of butterfly flight reveals strong coupling between membrane deformation and unsteady flow structures, including vortex formation and persistence beyond stroke reversal. These observations highlight that deformable membranes can actively shape local flow fields, motivating investigation of similar interactions when such membranes are placed, for example, within a rotor-induced flow, in the case of a hybrid flap-wing rotor bio-inspired micro air vehicle (BMAV).

#### 2.3.1. Image Pre-Processing

Table 3 presents the camera and schlieren settings for each recording analysed.

High-speed schlieren recordings were first converted to grayscale and stored in floating point format to ensure consistent numerical handling across the full image sequence. A geometric calibration was performed using a reference length to establish the spatial scale, and a fixed region of interest (ROI) was defined to isolate the schlieren test section and minimize boundary artifacts. Where necessary, frames were registered to a reference image to compensate for small camera or setup drifts. A background image acquired under identical optical conditions, but without the butterfly present, was used as a reference for subsequent processing.

To isolate flow- and body-induced refractive index variations, each frame was processed using background subtraction, either preserving the sign of the intensity difference or using the absolute difference depending on the analysis objective. Background subtraction is necessary in schlieren imaging to suppress stationary refractive index gradients arising from ambient convection and optical inhomogeneities, thereby enhancing flow structures generated exclusively by wing motion.

A mild Gaussian spatial filter (σ = 1.5 pixels, 3 × 3 kernel) was applied to suppress high-frequency sensor noise while preserving coherent schlieren gradient structures.

**Figure 7:**
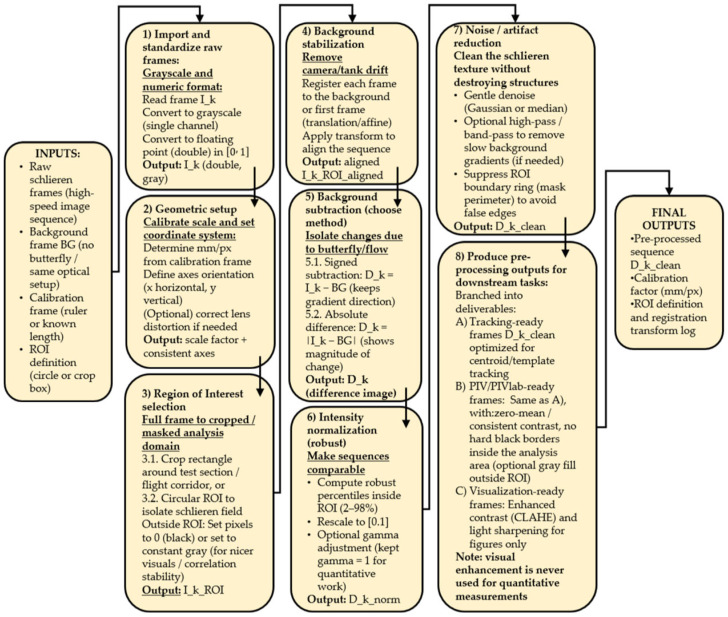
General diagram of the pre-processing steps. The arrows show the order of the pre-processing steps.

The resulting difference images were robustly normalized within the ROI to ensure consistent contrast across the sequence while limiting the influence of outliers. Gentle spatial filtering was applied to suppress sensor noise and residual optical texture without degrading coherent flow structures. The pre-processed images produced by this workflow form the basis for all subsequent analyses, including centroid tracking, kinematic reconstruction, and PIV-based visualization of vortex structures, while enhanced versions of the same frames were generated separately for qualitative visualization purposes. A clear diagram of the process is presented in Figure 7.

#### 2.3.2. Kinematic Extraction

##### Trajectory Reconstruction

Trajectory reconstruction is performed in MATLAB R2020a [37], for two different cases, first one is a vertical ascent flight and the second one is a diagonal flight on the schlieren mirror diameter. The process of obtaining the trajectory is described here, while the output is presented in the Results section.

The schlieren sequence is processed frame-by-frame using a background subtraction approach constrained to a circular region of interest (ROI) centred on the measurement zone. Each image is converted to grayscale and stored in floating point format, after which a circular mask is applied to suppress regions outside the optical test area. To improve robustness against illumination variations and schlieren texture, frames are mildly smoothed and then intensity-normalized within the ROI using a percentile-based rescaling (2–98%), which reduces the influence of outliers while preserving the dominant contrast of the butterfly and flow features. Percentile-based rescaling (2–98%) was preferred over classical min–max normalization or histogram equalization because it reduces sensitivity to extreme intensity outliers while preserving the relative gradient structure necessary for consistent flow-feature tracking across frames.

A background image acquired under identical optical conditions is pre-processed in the same way and subtracted from each frame using an absolute difference, producing a change map that highlights the butterfly silhouette and the strongest refractive index gradients associated with its motion.

The butterfly position is estimated by segmenting the difference image via an adaptive threshold computed from per-frame ROI statistics (mean plus a multiple of the standard deviation), followed by morphological cleanup (small object removal, closing, and hole filling) to obtain a coherent blob. The centroid of the detected blob is taken as the instantaneous body position; when multiple candidate blobs exist, the algorithm selects either the largest component at initialization or, subsequently, the component closest to the previous centroid to prevent jumps to noise. The time-ordered centroid coordinates are then used to reconstruct the two-dimensional flight trajectory by connecting successive positions, and translational velocity is computed from frame-to-frame centroid displacement using the known frame rate and spatial calibration factor. The resulting centroid sequence is used to reconstruct the trajectory, which is overlaid as a dotted path on the final pre-processed frame for visualization. Translational speed is computed from successive centroid displacements, accounting for occasional dropped detections by scaling the displacement by the actual frame gap. Pixel-based speeds are converted to physical units using the calibration factor (mm/px), and both the instantaneous speed–time history and the mean speed over the valid track are reported. Finally, centroid coordinates and a companion “value” metric (blob area in pixels) are exported to an Excel file to support further post-processing and quality control.

##### Membrane Deformation Tracking

Tracking membrane deformation during wing motion provides direct insight into fluid–structure interaction mechanisms that cannot be inferred from rigid kinematics alone. Changes in camber, cupping, and local curvature strongly influence the formation and persistence of unsteady flow structures, particularly in transitional and low-Reynolds-number regimes. The analysed schlieren sequences demonstrate the feasibility of extracting membrane outlines and deformation patterns at high temporal resolution, enabling phase-resolved analysis of wing behaviour. Beyond the current 2D representation, the experimental setup and data processing workflow are compatible with three-dimensional reconstruction using multiple viewing angles, allowing future extension to full 3D membrane micro-deformation tracking. Importantly, these techniques are applicable under natural lighting conditions, without reliance on laser-based illumination, supporting robust and scalable measurements.

Membrane deformation is extracted from the schlieren image sequence using a contour-based tracking approach built upon the same pre-processing framework employed for trajectory reconstruction. Each frame is converted to grayscale, restricted to the predefined region of interest, and normalized using a percentile-based rescaling to ensure consistent contrast. Background subtraction is then applied to enhance features associated with the wing and membrane, while suppressing stationary optical structures. Because schlieren imaging emphasizes refractive index gradients rather than solid boundaries, the membrane is identified using gradient-based feature detection rather than simple intensity thresholding.

Following pre-processing, edge information is extracted from the difference images to highlight the membrane boundary, and weak or isolated edge fragments are removed through light morphological filtering to improve spatial coherence. The resulting traced membrane deformation is presented in Results.

##### Body Positioning and Control

**Figure 8:**
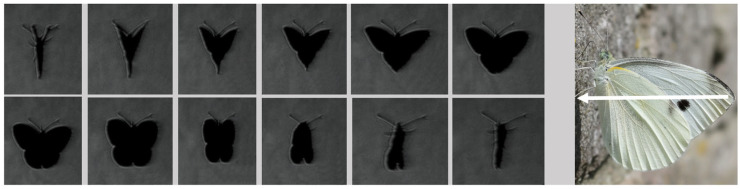
Schlieren image sequence showing a butterfly during vertical walking, with coordinated wing motion and surface contact (left). The image on the right illustrates the qualitative force exerted by the wing on the surface (arrow), emphasizing the supportive, non-aerodynamic role of the wings. [*Pieris rapae* shot in both schlieren and natural light, in free vertical walk].

Qualitative observations of butterfly behaviour during ground contact further reinforce the multifunctional role of membrane wings beyond aerodynamic lift. When walking on the schlieren mirror, the butterfly uses its wings to apply normal force against the surface, contributing to body stabilization and posture control through compliant deformation rather than flapping. This behaviour highlights the ability of membrane wings to act as mechanically adaptive interfaces with nearby structures, distributing loads and generating stabilizing moments. The emphasis on this wing motion is on the cupping at the top (as depicted in Figure 8), which, according to previous studies, produces thrust, while down stroking produces lift. On the schlieren recording, the cupping effect can be easily observed as a movement of the thermal gradients present in the laboratory environment. Such non-propulsive wing functions are directly relevant to future BMAV architectures, where wings are intended to support stability, disturbance damping, and interaction in close proximity to surfaces (e.g., during docking or stand-by).

In addition to membrane deformation, preliminary tracking of body and wing trajectories demonstrates the feasibility of extracting kinematic information relevant to posture control and motion stabilization, without implying aerodynamic performance.

Rapid abdominal reorientation is quantified from selected schlieren frames using a manual, geometry-based angular measurement procedure designed to remain robust in the presence of strong optical gradients and complex flow structures. For each analysed frame, two reference lines are defined directly on the image by manual selection of four points: two points corresponding to the longitudinal axis of the thorax and two points corresponding to the principal axis of the abdomen. These points are used to construct direction vectors representing the instantaneous orientation of each body segment within the image plane.

The relative angle between the thorax and abdomen vectors is computed using a dot product formulation. Both the acute and obtuse angles between the two vectors are evaluated, and the larger angle is retained as the representative metric in order to capture the full extent of abdominal reorientation during large-amplitude manoeuvres. Each measurement is visually documented by overlaying the selected points, line segments, and computed angle values onto the original schlieren image, which is saved for traceability and qualitative validation. This approach enables time-resolved quantification of rapid abdominal orientation changes over successive frames within a single wingbeat cycle, forming the basis for the kinematic analysis presented in the Results section.

##### Fling Motion

**Figure 9:**
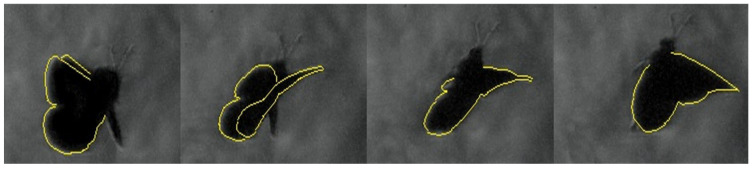
Schlieren images illustrating a slow lateral S-like wing-fling motion used by a butterfly to interact with weak warm-air convection currents, resulting in enhanced buoyant support through passive flow coupling. The contour of the wings is displayed in yellow.

Preliminary schlieren observations show that butterfly wings can couple effectively to weak convective flows through a slow lateral “fling” motion (illustrated in Figure 9) when a thermal gradient is present. Unlike rapid flapping, this motion involves large deformation at low frequency and amplitude, indicating a low energy cost while still producing a noticeable interaction with the surrounding flow. In several sequences, even weak warm air currents were sufficient to modify body motion when combined with this slow wing movement. These observations suggest that flexible membrane wings can serve as energy-efficient flow-interaction surfaces, capable of exploiting ambient thermal disturbances with minimal actuation effort.

Thermal structures were identified from background-subtracted schlieren intensity maps, where localized refractive index gradients originating near the abdomen were tracked over consecutive frames. The analysis is qualitative and based on relative gradient magnitude rather than absolute temperature measurement.

The post-processing algorithm used here is similar to the one used for tracing the membrane.

This motivates consideration of an additional wing function for BMAVs, focused on low-power interaction with naturally occurring unsteady or thermally driven flows.

##### Wake and Vortex Identification

**Figure 10:**
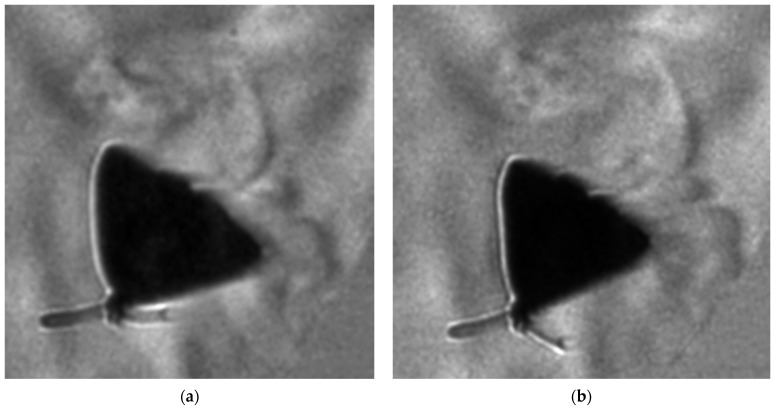
Flow structures near wing tip (consecutive frames (**a**,**b**)).

**Figure 11:**
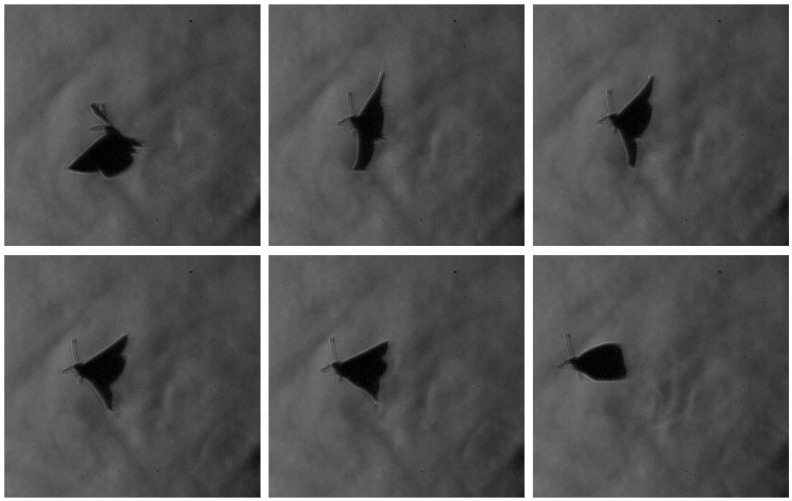
Stills highlighting transient force augmentation and manoeuvring capability.

High-speed schlieren imaging reveals the formation of coherent rotational flow structures near the wing tips when the wings approach each other in the upper clap position. These structures appear as localized regions of curved refractive index gradients, persisting over multiple frames and convecting away from the wing tip following wing contact (Figure 10). A comparison of pre- and post-clap kinematics indicates an approximate 30% increase in translational speed following the upper clap event, observed without a concurrent change in body orientation (Figure 11), suggesting that the clap/fling sequence can contribute to transient force augmentation and manoeuvring capability—an effect of direct relevance for the design of bio-inspired micro air vehicle propulsion strategies. The mean centroid velocity increased from 1.20 ± 0.08 m/s (n = 45 frames) before the clap event to 1.56 ± 0.10 m/s (n = 48 frames) immediately after the clap interaction, corresponding to the approximate 30% increase in translational speed.

**Figure 12:**
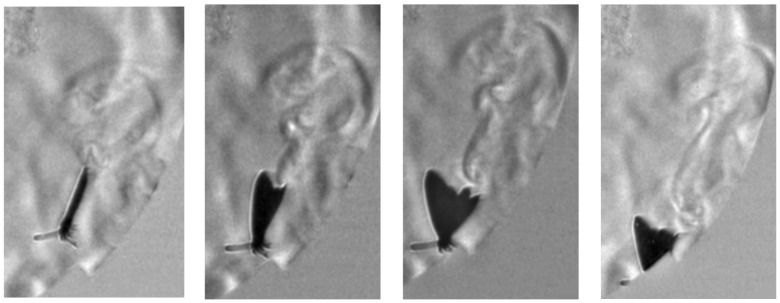
Post-processed schlieren images revealing butterfly’s thermal signature, generated by the abdomen and distributed by the wing area.

Figure 12 illustrates the formation of a vortex associated with the clap phase of the wingbeat cycle. The vortex is clearly visible owing to the enhanced sensitivity of the schlieren system to localized refractive index gradients. In addition, a transient increase in the butterfly’s core temperature contributes to the visibility of the structure, as evidenced by the intensified schlieren signatures observed in the second and third frames.

#### 2.3.3. Other Important Qualitative Observations

The paper further presents a visualization of the beginning of a butterfly’s downstroke for the Pieris rapae subject, recorded in free flight conditions, in vertical (ascending flight). Butterflies primarily use the downstroke for generating weight support and the upstroke for generating thrust, as described by [36].

**Figure 13:**
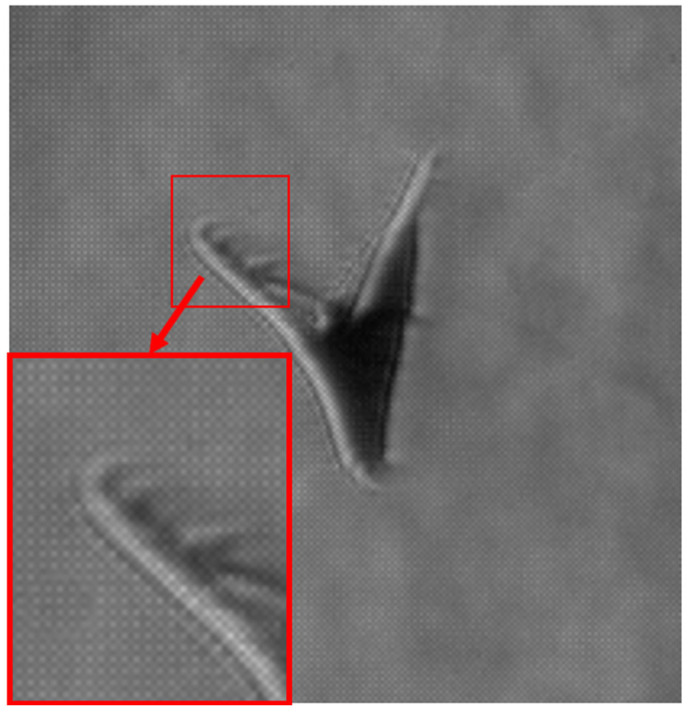
Zoomed-in schlieren image (red frame) showing air entrained between the wings at the end of the upstroke. The localized structure appears confined between the wing surfaces near their uppermost position and is indicative of cupped air during the clap phase.

The zoomed-in schlieren image highlights a localized flow structure forming between the wings as the butterfly reaches the start of the downstroke (Figure 13). At this phase of the wingbeat cycle, the wings start to pull away from their uppermost position prior to separation, corresponding to the clap phase of flight. The detailed visualization reveals a confined region of refractive index variation located between the wing surfaces, consistent with a volume of air being temporarily attached to the wing. Because this structure appears spatially coincident with the wing membranes in a single frame, it could be mistakenly interpreted as a deformation of the wing itself.

**Figure 14:**
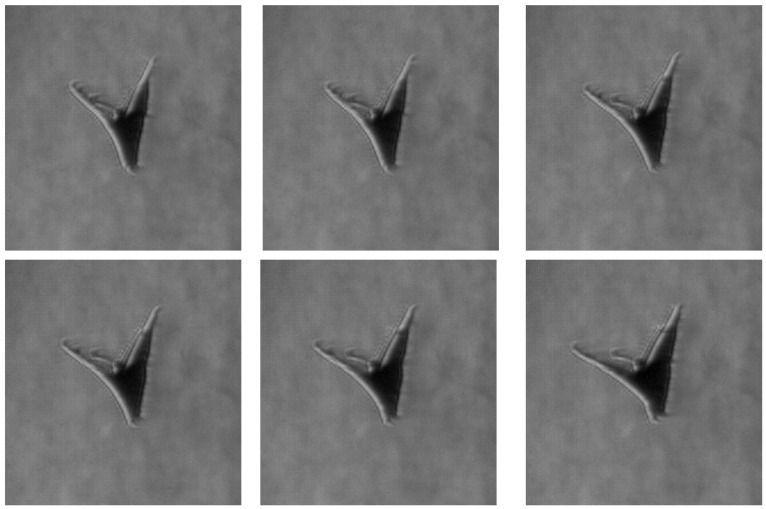
High-speed schlieren image sequence showing flow structures forming between the wings near the end of the upstroke. A localized refractive index variation appears when the wings reach their uppermost position and is initially confined between the wing surfaces. As the wings begin to separate, this structure detaches and spreads into the surrounding flow, rolling into small-scale vortical features with a rotation opposite to the local wing motion. The temporal evolution observed across successive frames indicates that the structure corresponds to entrained air released during the clap phase rather than to elastic deformation of the wing membrane.

Examination of the full image sequence (Figure 14) clarifies the nature of this feature. As the wings begin to separate from their uppermost position and transition into the early downstroke (fling phase), the structure detaches from the wing surface and spreads into the surrounding flow, rolling up into small-scale vortical features. The direction of this spreading and rotation is opposite to the local wing motion on which the structure initially formed, behaviour that is inconsistent with a purely elastic membrane response. Instead, the observed evolution is characteristic of entrained air released during wing separation and reorganized into vortical motion, supporting the interpretation that the schlieren signatures correspond to cupped air associated with the clap–fling mechanism rather than to structural wing deformation.

#### 2.3.4. Non-Dimensional Metrics

In the context of the present study, the Strouhal number (St) is used to characterize the unsteady wing kinematics of the butterfly under different flight conditions, including hovering and plume-assisted ascent. It is defined by the Equation (4).

(4) $$St=\frac{fA}{U}$$

where *f* is the wingbeat frequency, *A* is the peak-to-peak wingtip stroke amplitude, and *U* is the characteristic body velocity. For cases involving hovering or plume riding, the ascent velocity is used as the reference velocity *U*, as it represents the dominant translational motion of the butterfly relative to the surrounding air. This formulation enables direct comparison between steady forward flight and vertically dominated flight regimes, and provides a consistent non-dimensional framework for assessing the role of clap-and-fling kinematics in force generation and manoeuvring.

The averaged St number is computed in this paper for the next cases: (a) vertical ascent, (b) thermal plume riding and (c) hovering.

(a) Vertical ascent—for this flight condition, the average ascent velocity is extracted from the image sequence by tracking the insect centroid on a frame-by-frame basis. Spatial calibration is performed using known physical dimensions, namely the schlieren mirror diameter and a reference cylinder of 2 mm diameter, which are correlated with their corresponding pixel measurements in the images. The wingbeat frequency is determined over ten consecutive cycles, while the stroke amplitude is obtained by tracking the wingtip motion over the same cycles. Wingbeat frequency is evaluated using two independent approaches: first, by applying a kymograph-based analysis implemented in ImageJ.JS 1.54m [38], and second, through manual verification of the periodicity in the image sequence.

(b) Thermal riding—for the thermal riding condition, the butterfly motion is characterized by a slow, predominantly vertical displacement induced by interaction with an ambient thermal plume. The reference velocity is extracted from the image sequence by tracking the insect centroid on a frame-by-frame basis and computing the mean ascent rate over the selected interval. Spatial calibration is performed using the same reference dimensions as in the vertical ascent case to ensure consistency across flight regimes. Wingbeat frequency is determined over ten consecutive cycles using the kymograph-based method implemented in ImageJ.js [38] and verified manually, while the stroke amplitude is obtained by tracking the wingtip motion over the same cycles.

(c) Hovering—for hovering conditions, the butterfly exhibits minimal net translational motion, with small oscillatory displacements about a nearly fixed position. The reference velocity is therefore defined as the mean vertical velocity computed from centroid tracking over the analyzed time window. Spatial calibration follows the same procedure as for the other flight regimes.

In the present study, the Strouhal number provides a compact, non-dimensional framework for comparing wingbeat kinematics across three distinct flight regimes—vertical ascent, thermal riding, and hovering—despite their markedly different translational characteristics. By relating wingbeat frequency and stroke amplitude to a characteristic body velocity, the Strouhal number enables direct assessment of how unsteady wing motion scales with the effective motion of the butterfly relative to the surrounding air. For vertical ascent, the ascent velocity represents the dominant translational component, while for thermal riding and hovering the reference velocity is defined by the mean vertical displacement induced by plume interaction or residual body motion. Using a consistent Strouhal formulation across these cases allows changes in wing kinematics, rather than absolute speed alone, to be highlighted and compared. In this context, variations in Strouhal number reflect adjustments in the clap–fling dynamics and force modulation strategy adopted by the butterfly under different aerodynamic and environmental conditions, providing insights that are directly relevant for the design and control of bio-inspired micro air vehicles operating across multiple flight modes.

### 2.4. Additive Manufacturing and FEA Simulation

A CAD model has been designed for the butterfly wings as the first iteration in the direction of prototype development. The model is inspired by the wings of the species *Idea leuconoe* as described in [39]. The models were manufactured through AM on the Form3+ [40] printer, using Tough2000 resin [41].

The CAD geometry was derived from *Idea leuconoe* due to the availability of detailed morphometric and venation data in the literature, enabling accurate proportional modelling without requiring invasive measurement of the experimentally observed specimens (*Iphiclides podalirius* and *Pieris rapae*); the prototype therefore represents a structurally bio-inspired abstraction rather than a direct replica of the imaged butterflies.

**Figure 15:**
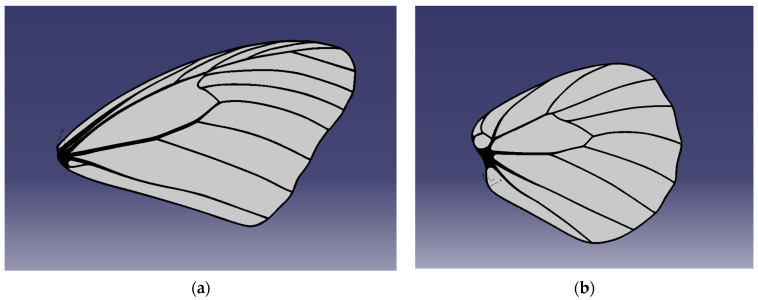
CAD design of the (**a**) forewing and (**b**) hindwing.

Figure 15 presents the 3D cad model of the forewing and hindwing. The entire wing has been 3D printed from the same material, the membrane had a thickness of 0.5 mm, while the venation model has a thickness of 1.5 mm. The resulted printed models are presented in the Results section, together with the optimized printing parameters and two printing iterations.

An FEA analysis was also conducted on the physical model to extract the normal modes of the newly manufactured wings (model illustrated in Figure 15).

## 3. Results

### 3.1. Image-Based Quantitative Results

While Section 2.3 introduced the impulse-based force framework for completeness, the quantitative results presented in this section focus on kinematic reconstruction and non-dimensional metrics derived from image-based tracking, rather than direct wake force integration. The analysed schlieren sequences cover multiple flight regimes, including diagonal forward flight, controlled vertical ascent, thermal plume-assisted ascent, near hovering, extreme manoeuvring (inversion–righting), and wing-assisted ground stabilization.

#### 3.1.1. Membrane Deformation Tracking

**Figure 16:**
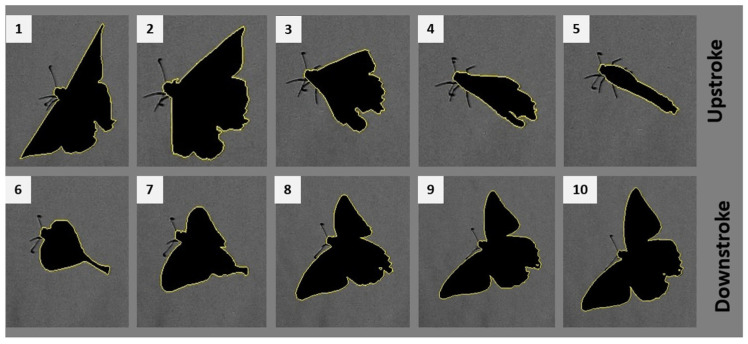
Representative high-speed schlieren frames of a butterfly wingbeat, showing a both partial upstroke (frames 1–5) and downstroke (frames 6–10). Extracted membrane outlines (yellow) highlight phase-dependent wing deformation and cupping. Images are shown for qualitative illustration of membrane tracking capability (*Iphiclides podalirius* shot in free flight).

The membrane contour is subsequently identified as a continuous boundary in the vicinity of the wing region, with spatial constraints applied to exclude unrelated flow structures (Figure 16). Temporal coherence is enforced by propagating the membrane contour from one frame to the next, allowing only limited local displacements between consecutive frames. This prevents the contour from jumping to nearby schlieren features unrelated to the wing. The resulting tracked membrane contours provide a time-resolved description of wing deformation, from which quantities such as camber evolution, local curvature, and cupping depth can be derived over the wingbeat cycle. Phase-averaged membrane shapes are obtained by synchronizing the tracked contours with the wingbeat frequency, enabling direct comparison between membrane deformation and the observed flow structures during clap-and-fling motion.

#### 3.1.2. Free Flight Kinematics and Trajectories

**Figure 17:**
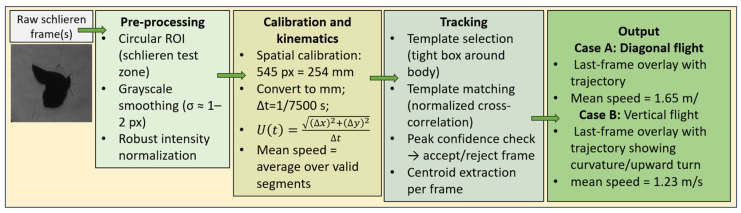
Processing scheme of the butterfly diagonal and vertical flight.

The trajectory reconstruction algorithm has been applied to two different sequences, as described in Figure 17.

**Figure 18:**
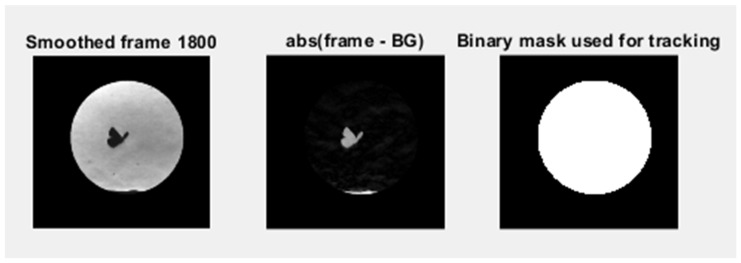
Pre-processing of the initial diagonal flight frames as resulted from MATLAB R2020a.

**Figure 19:**
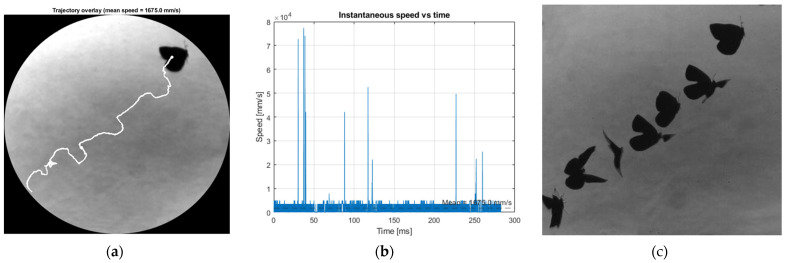
(**a**) Trajectory resulted by centroid tracking for the diagonal flight, (**b**) mean speed graph calculated from centroid tracking as a function of time, and (**c**) overlay of the butterfly position in time.

**Figure 20:**
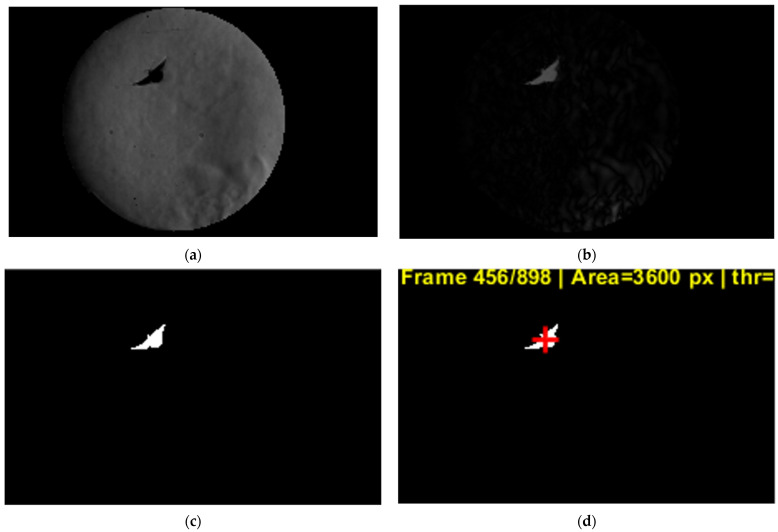
Phases of the vertical flight frames processing: (**a**) Pre-processed frame with background subtraction and applied circular mas, (**b**) object identification through pixel intensity, (**c**) threshold applied for better object identification, (**d**) centroid (red cross) tracking.

**Figure 21:**
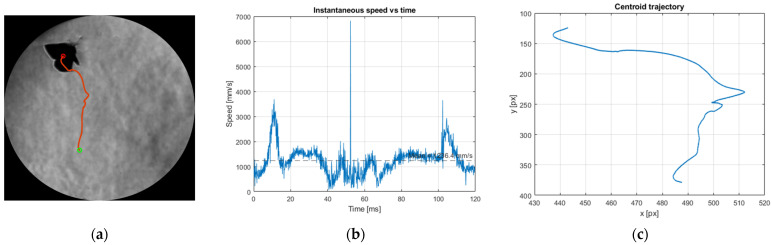
Centroid-based kinematic reconstruction for a representative vertical flight sequence: (**a**) Trajectory overlaid on the final schlieren frame, obtained from frame-by-frame centroid tracking within the circular region of interest. (**b**) Instantaneous ascent speed as a function of time, with the mean speed of 1236.4 mm/s indicated. Short-duration spikes reflect transient centroid estimation uncertainty during rapid wing motion. (**c**) Reconstructed centroid trajectory in the image plane, illustrating predominantly vertical displacement consistent with vertical ascent.

The results of processing the two image batches according to the processing steps described in Section 2 are illustrated below in Figure 18 and Figure 19 for diagonal flight and Figure 20 and Figure 21—for vertical flight.

The centroid-based reconstruction reveals a coherent diagonal flight trajectory with a well-defined mean translational velocity of approximately 1675 mm/s (1.675 m/s). This value is consistent with the visual displacement observed across the schlieren field of view over the analysed time interval and indicates an actively propelled flight segment rather than passive drifting or plume-assisted motion. The trajectory overlay and time-stacked butterfly positions confirm that the tracking procedure captures the overall motion reliably, providing confidence in the extracted mean velocity as a representative descriptor of the butterfly’s translational dynamics during this manoeuvre. The instantaneous speed history derived from frame-to-frame centroid displacements exhibits several short-duration spikes that locally exceed the baseline speed. These fluctuations are attributed to transient uncertainties in centroid estimation caused by rapid wing motion, partial silhouette overlap during clap–fling phases, and segmentation ambiguities inherent to schlieren imaging of deforming bodies. Importantly, these elevated values are isolated in time and do not persist over consecutive frames, indicating that they do not correspond to sustained physical accelerations. As a result, their influence on the time-averaged velocity is limited, and the reported mean speed remains a robust and physically meaningful measure for comparison with other flight regimes examined in this study.

The centroid-based analysis for this case indicates a predominantly vertical flight trajectory, with limited lateral displacement, as confirmed by both the trajectory overlay and the reconstructed centroid path. The mean translational velocity extracted from this sequence is approximately 1236.4 mm/s (1.24 m/s), which is lower than the value obtained for the diagonal flight case and is consistent with a vertically oriented ascent rather than an aggressively propelled manoeuvre. The reconstructed trajectory shows sustained upward motion, indicating that this sequence corresponds to a controlled vertical flight regime.

The instantaneous speed history exhibits moderate fluctuations about the mean value, with several short-duration peaks exceeding the baseline speed. As in the previously analysed cases, these peaks are attributed to transient uncertainties in centroid localization arising from rapid wing motion, partial silhouette overlap, and segmentation variability inherent to schlieren imaging. These elevated values are isolated in time and do not persist across consecutive frames, indicating that they do not represent sustained physical accelerations. Consequently, the mean ascent velocity is retained as a robust reference value and is used in the subsequent Strouhal number evaluation, enabling direct comparison between vertical ascent and other flight regimes examined in this study.

#### 3.1.3. Strouhal Number for Vertical Ascent, Thermal Riding and Hovering

In the next section, the Strouhal number is computed for the vertical ascent case.

**Figure 22:**
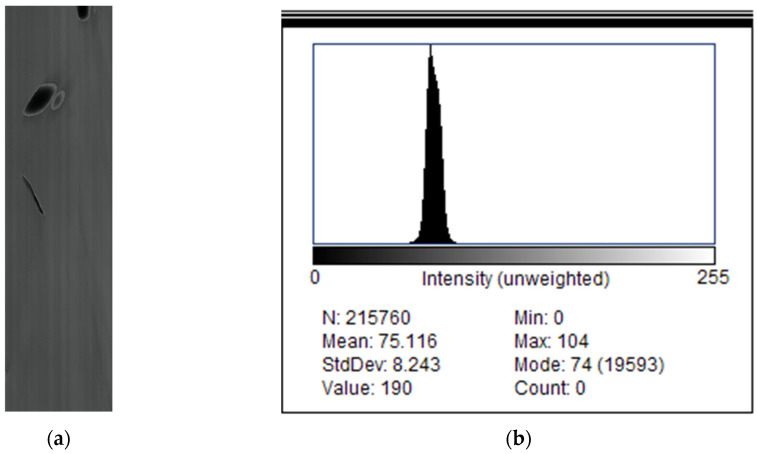
(**a**) Kymograph of the vertical ascent flight and (**b**) histogram of the kymograph identifying the kymograph intensity peaks.

Due to slight lateral drift and non-planar wing motion during flight, the wing intersects the kymograph (Figure 22) sampling line at multiple spanwise locations within a single cycle, resulting in multiple streaks per wingbeat. While this limits the use of the kymograph for precise spatial amplitude extraction, the temporal periodicity remains clearly identifiable and was therefore used to determine the wingbeat frequency, which was subsequently verified by manual inspection of the image sequence. The kymograph obtained is based on a batch composed of 899 consecutive schlieren frames.

The resulting wingbeat frequency has been calculated considering the real recording rate of 7518 fps. The wingbeat frequency was computed as the mean over ten consecutive cycles. Based on cycle-to-cycle variation, the estimated uncertainty is approximately ±0.2 Hz, yielding a final reported value of 13.3 ± 0.2 Hz.

Butterflies as a group typically exhibit relatively low wingbeat frequencies compared with smaller insects such as flies or bees. Broad surveys and comparative studies show that lepidopterans (the order that includes butterflies and moths) can span a wide range of frequencies, generally from the single digits up to several tens of hertz, depending on species and size. For example, one study of wingbeat frequencies across insect taxa reports that lepidopterans (butterflies and moths) range broadly from about 6.7 Hz up to over 80 Hz, reflecting significant inter-specific differences in size and flight style [42]. More specifically, descriptive accounts note that butterfly wingbeat frequencies are generally around 10 Hz, which is among the lowest among flying insects due to their large wing area and relatively slow but powerful strokes [31]. The amplitude was obtained by tracking the wingtip position across 10 cycles and averaging the result. The wingtip position was manually checked as well for improving accuracy.

A Strouhal number of 0.18 was obtained for the present vertical flight. This value falls within the lower end of the biologically relevant range typically reported for efficient flapping flight (St≈0.2–0.4) [43]. This value is consistent with a controlled vertical flight regime characterized by moderate stroke amplitude and relatively steady ascent velocity, rather than aggressive manoeuvring or hovering. The result supports the physical consistency of the extracted kinematics and indicates that the butterfly operates close to the optimal Strouhal regime even during non-horizontal flight.

Across the analysed flight sequences, the wingbeat frequency varied within approximately 13–16 Hz, remaining within the lower-frequency regime characteristic of butterfly flight reported in the literature.

The Strouhal number associated with the thermal fling segment was estimated at approximately 0.56, exceeding the classical efficient cruising range. This elevated value reflects the large stroke amplitude and unsteady vortex-dominated nature of the clap–fling motion, which prioritizes transient force generation over steady propulsive efficiency.

For the thermal riding case, the Strouhal number was calculated using the mean vertical centroid velocity as the reference speed, averaged over the selected time window to reduce sensitivity to short-term fluctuations.

In the near-hover regime, the net translational velocity of the butterfly is extremely small and fluctuates around zero over the analysed time window. Because the Strouhal number is defined as St=fA/U, its evaluation becomes highly sensitive and physically ill-conditioned as the reference velocity U approaches zero. Small uncertainties in centroid tracking or short-term drift can therefore produce disproportionately large or unstable Strouhal values that do not meaningfully reflect the underlying aerodynamics. For this reason, the Strouhal number is not reported for the near-hover case. Instead, the kinematic behavior is suggested to be characterized directly through wingbeat frequency and stroke amplitude, which in this flight regime provide a more robust description of the flapping dynamics under quasi-hovering conditions.

#### 3.1.4. Thorax-Driven Manoeuvring and Directional Control

**Figure 23:**
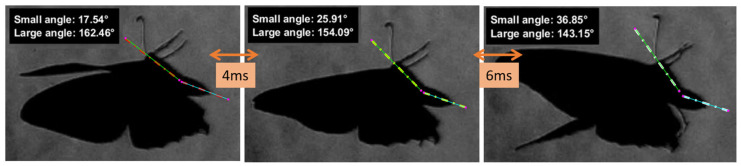
High-speed schlieren frames illustrating abdomen motion and control during extreme manoeuvring flight. Dashed lines indicate the extracted abdomen orientation, with measured small and large inter-segment angles shown for each frame. The frames are separated by approximately 4 ms and 6 ms, corresponding to closely spaced phases within a wingbeat at an effective frequency of ≈15–16 Hz. The pronounced change in abdomen orientation over short time intervals highlights active body reconfiguration associated with aggressive manoeuvring.

High-speed schlieren observations reveal pronounced, rapid abdomen reorientation during aggressive butterfly manoeuvres, occurring over only a few milliseconds within a single wingbeat cycle (≈15–16 Hz), as presented in Figure 23. The measured angular changes indicate that the abdomen acts as an active control element, contributing to attitude regulation, inertia redistribution, and transient stabilization during non-steady flight. While the present analysis does not yet replicate abdominal control precisely, these observations are highly relevant for future BMAV architecture, where control authority is similarly expected to arise from low-mass, deformable elements interacting with unsteady flow rather than from continuous propulsion changes. The butterfly abdomen swing therefore provides a biological analogue for distributed, non-propulsive control strategies, supporting the investigation of membrane-based surfaces as effective contributors to the manoeuvring and stability of aerial systems operating under disturbed or transient conditions.

The observed rapid abdomen reorientation suggests a bio-inspired pathway for disturbance rejection via inertia redistribution. In future work, this could be formalized as a learning-based control augmentation (trained in simulation and validated experimentally) that maps local flow and inertial cues to stabilizing moment commands while retaining a conventional baseline controller for safety.

#### 3.1.5. Inversion and Righting Manoeuvre

**Figure 24:**
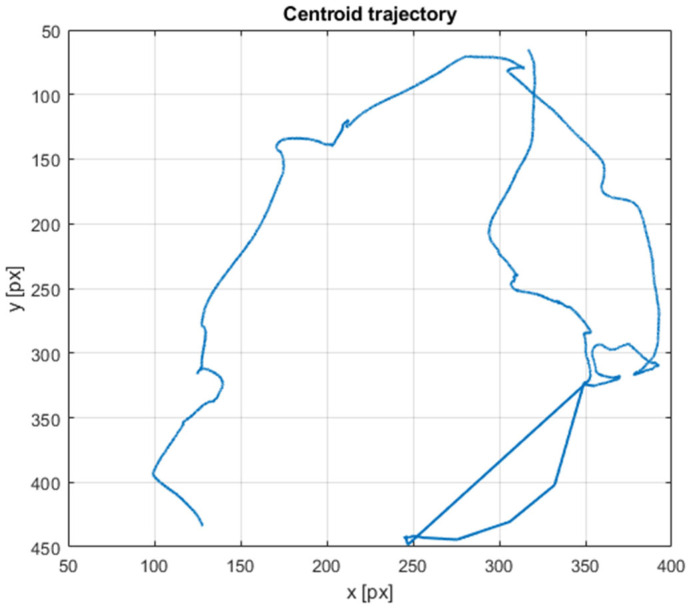
Extreme manoeuvring trajectory.

A representative sequence capturing a transient inverted flight condition followed by rapid righting is presented as an extreme manoeuvre case study. The flight trajectory for this case is reconstructed through the centroid tracking method, presented in Section 3.1.2. This is represented in Figure 24 in order to highlight the extreme manoeuvring motion needed to correct the inverted flight.

In the initial frames, the butterfly is observed in an inverted or near-inverted orientation, with the dorsal surface facing downward and the body axis misaligned with the nominal flight direction. Schlieren visualization highlights pronounced flow asymmetry during this phase, particularly near the wingtip regions.

**Figure 25:**
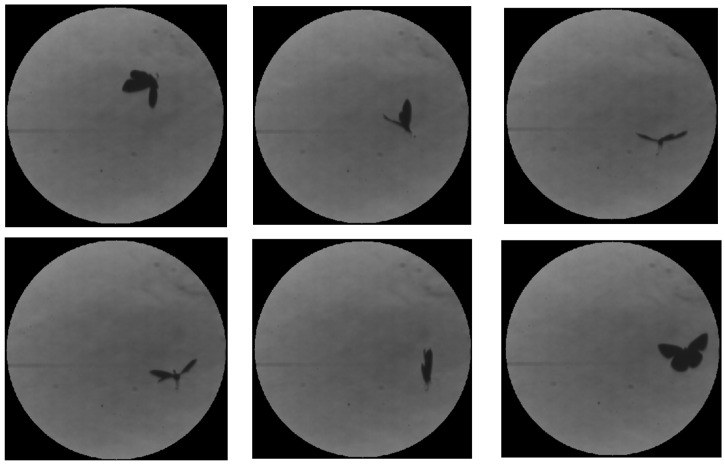
Extreme manoeuvring of the butterfly inverted flight redress.

The temporal evolution of the body angle reveals a rapid reorientation occurring within a fraction of a wingbeat cycle (schlieren images from the sequence presented in Figure 25). Angular measurements extracted from successive frames indicate a substantial change in body pitch, confirming that the righting manoeuvre is not gradual but instead occurs through a short-duration, high-amplitude adjustment. The associated flow structures exhibit strong asymmetry, with intensified tip vortex signatures forming preferentially on one side. This imbalance suggests differential force generation between the two wings during the manoeuvre.

Notably, the thorax motion appears to act as the primary driver of the righting mechanism. Rather than relying solely on symmetric wingstroke modification, the butterfly executes pronounced thoracic swings that redistribute inertia and alter the effective aerodynamic loading. The combination of asymmetric vortex formation and rapid thorax reorientation supports the interpretation that body segment kinematics contribute significantly to transient attitude control. This extreme manoeuvre highlights the capacity of the butterfly to achieve rapid stabilization through coordinated aero–inertial coupling, offering insight into non-propulsive control strategies relevant to bio-inspired micro air vehicle design.

### 3.2. Additive Manufacturing of the Future Prototype Wings

The butterfly wing replicas were fabricated using a Form 3+ stereolithography (SLA) printer and Tough 2000 resin for both the venation network and the membrane layer. The wing geometry followed a bio-faithful venation layout derived from morphological observations, which resulted in a highly slender and delicate structural network. Due to the thin membrane and fine venation features, support placement required careful manual optimization within the slicing software to minimize stress concentrations and avoid direct contact with the most compliant membrane regions. Despite these precautions, support removal remained delicate, particularly in areas of dense venation, where mechanical interaction could induce localized deformation. The selected layer thickness was 100 µm. Laser exposure energy and printing speed were controlled automatically by the PreForm [44] software according to the validated resin profile.

Post-processing was adapted to preserve membrane integrity. The standard automated washing module was not used, as agitation during the washing cycle caused membrane distortion. Instead, printed parts were immersed statically in a container of isopropyl alcohol (IPA) for approximately 30 min without agitation to allow residual uncured resin to dissolve gradually. Following cleaning, the parts were air-dried and subjected to a brief post-curing cycle not exceeding 5 min. Extended curing times were avoided because they increased material brittleness and reduced membrane compliance. This modified post-processing protocol enabled retention of the intended flexible behaviour of the thin membrane while maintaining sufficient structural stiffness in the venation network.

**Figure 26:**
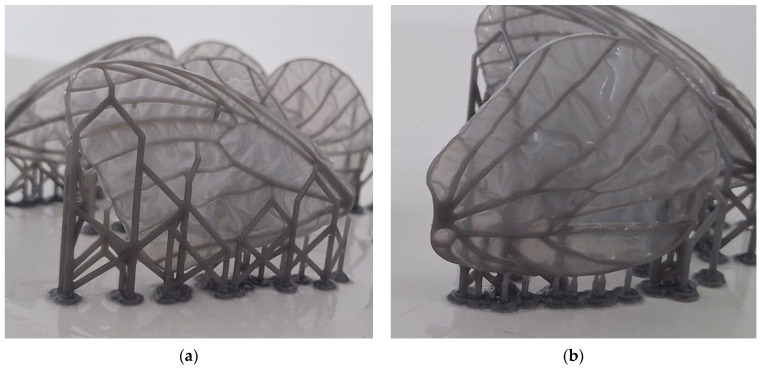
3D-printed tough resin butterfly wings with deformed membrane: (**a**) Forewing and (**b**) hindwing.

The iteration of the butterfly wings run through Form Wash [45] software, for which the membrane suffered deformation, is presented in Figure 26.

**Figure 27:**
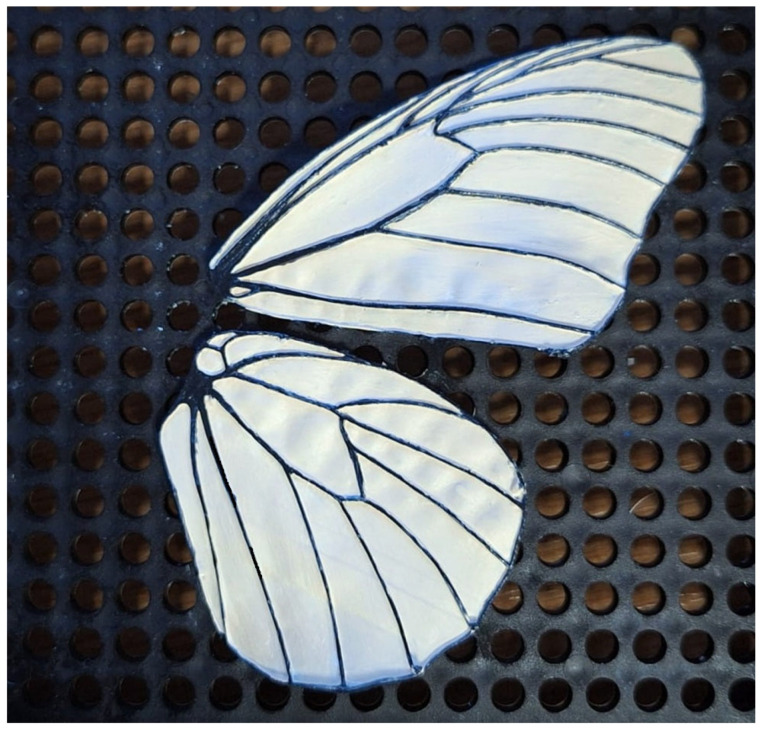
White painted membrane bio-inspired butterfly wing prototypes manufactured following optimization of the stereolithography printing and post-processing workflow.

Following iterative optimization of the printing parameters and post-processing procedure, a noticeable improvement was achieved in the geometric fidelity and structural integrity of the fabricated wings, as exemplified in Figure 27. Based on the outcomes of this manufacturing iteration loop, the final prototype wings will be produced using the material configuration that demonstrated the most favourable balance between membrane compliance and venation stiffness.

### 3.3. FEA

Normal mode analyses of the butterfly wings are performed to evaluate their dynamic response to aerodynamic loads in the frequency domain. These analyses provide insight into the natural vibration characteristics of the wings, which are important for understanding their stability, flexibility, and potential aeroelastic behaviour during flapping or gliding.

The analyses are conducted using a free–free finite element model (FEM). In a free–free finite element model, no boundary constraints are imposed on the structure, meaning that it is unconstrained in space and the first six modes correspond to rigid body translations and rotations with near-zero natural frequencies. Under these boundary conditions, the first six vibration modes correspond to rigid body motions and therefore exhibit near-zero natural frequencies. The free–free boundary condition was intentionally selected to characterize the intrinsic dynamic properties of the additively manufactured wing independent of any specific root constraint or actuation architecture. At the present prototype development stage, a physically validated hinge mechanism and corresponding rotational stiffness parameters are not yet defined. Applying cantilever or hinged boundary conditions would therefore introduce assumed constraint stiffness values without experimental validation. The current analysis thus provides baseline structural eigenmodes of the wing geometry and material system, which will serve as a reference for future studies incorporating experimentally informed root attachment conditions.

**Figure 28:**
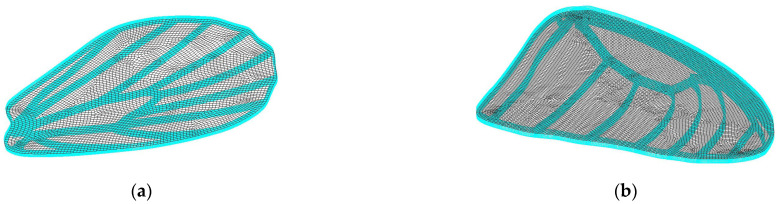
Representation of the finite element models: (**a**) Forewing and (**b**) hindwing. The venation pattern is depicted in cyan.

The study considers two butterfly wings, referred to as the forewing and hindwing. Their finite element models are illustrated in Figure 28. Structural ribs are discretized using hexahedral elements, while the wing membrane is modelled with shell elements to capture bending and stretching behaviour. The entire structure is assumed to be made of Tough 2000 Resin, with a density of 1090 kg/m^3^, Young’s modulus of 1.8 GPa, and Poisson’s ratio of 0.34, representing a lightweight but stiff material suitable for bio-inspired wing structures.

**Figure 29:**
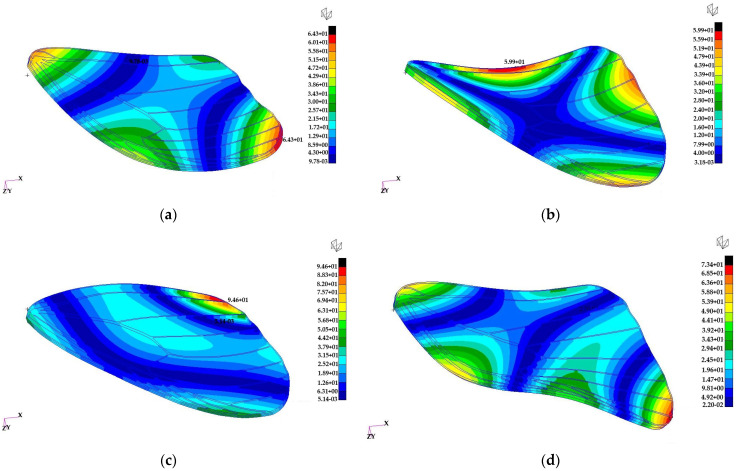
Forewing—normal mode eigenvector: (**a**) 1st bending mode eigenvector; (**b**) 2nd bending mode eigenvector; (**c**) 3rd bending mode eigenvector; and (**d**) 4th bending mode eigenvector.

The first four normal mode eigenvectors of the large butterfly wing are presented in Figure 29, highlighting the dominant deformation patterns.

**Figure 30:**
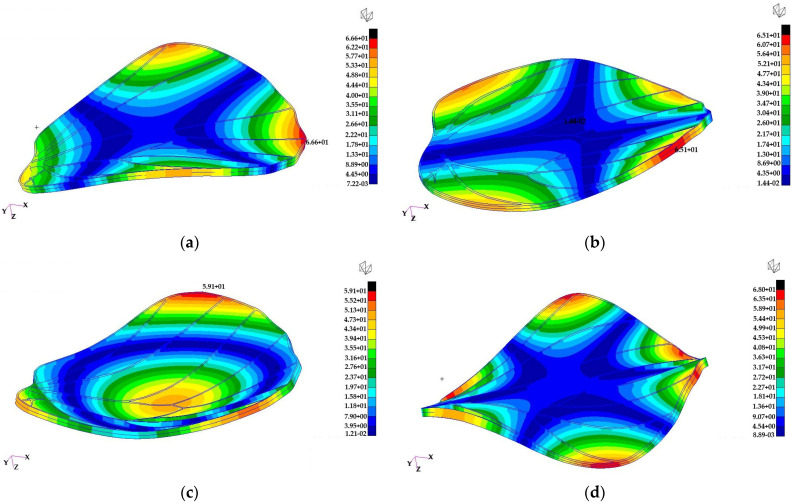
Hindwing—normal mode eigenvector: (**a**) 1st bending mode eigenvector; (**b**) 2nd bending mode eigenvector; (**c**) 3rd bending mode eigenvector; and (**d**) 4th bending mode eigenvector.

The bending mode eigenvectors of the small butterfly wing are shown in Figure 30, illustrating the primary flexural responses of the wing structure.

The wing eigenvectors can be compared with the normal mode eigenvectors of a circular plate to provide insight into the deformation characteristics:

• The first bending eigenvector resembles the (1, 1) mode of a circular plate.
• The second bending eigenvector resembles the (2, 1) mode of a circular plate.
• The third bending eigenvector resembles the (0, 1) mode of a circular plate.
• The fourth bending eigenvector resembles the (3, 1) mode of a circular plate.

These comparisons indicate that the wings exhibit bending patterns similar to classical plate modes, with higher-order modes showing increasing nodal lines and complexity. Such modal information is useful for designing bio-inspired flapping wings and understanding their response to dynamic aerodynamic loads.

## 4. Discussion

### 4.1. Wing–Body Coupling in Butterfly Flight

The present schlieren observations reinforce the hypothesis that butterfly flight cannot be interpreted as a purely wing-driven aerodynamic system, but rather as a strongly coupled aero–inertial mechanism in which thorax and abdomen motions contribute directly to manoeuvring and stabilization. The rapid abdominal reorientations and thoracic swings observed during aggressive manoeuvres indicate that inertia redistribution occurs within a fraction of a wingbeat cycle, modifying the effective loading of the wings and the wake topology. This finding aligns with prior theoretical frameworks describing body–wing coupling in insect flight [1,14], but extends them by providing direct visual evidence of flow under free-flight conditions.

Although size scaling in insect flight influences multiple coupled aerodynamic and structural parameters, including Reynolds number, structural stiffness, and mass distribution, the present analysis focuses on kinematically accessible quantities derived from high-speed imaging.

Unlike many dipteran models, which often approximate the body as a rigid fuselage with minor dynamic influence, the butterflies analysed here exhibit substantial body-axis excursions correlated with asymmetric vortex formation and transient force augmentation. In particular, the inversion–righting sequence demonstrates that thoracic motion acts as a primary control driver rather than a passive response. This suggests that, for low-frequency, large-amplitude flappers such as butterflies, attitude control may rely more heavily on body segment inertia and aeroelastic redistribution than on subtle wing stroke asymmetries alone.

### 4.2. Aerodynamic Role of the Upstroke

A central observation of this study is that the upstroke is not aerodynamically passive. Schlieren sequences show entrained air confined between the wings at the end of the upstroke, which subsequently detaches and rolls into coherent vortical structures during the fling phase. This behaviour is consistent with the classical clap–fling mechanism described by Weis-Fogh [15,16,17], yet the present recordings provide direct visualization of cupped air release and subsequent vortex roll-up in a freely flying butterfly.

Moreover, the identification of attached flow structures on cupped wings during the upstroke suggests that membrane deformation actively shapes local shear layers and vortex persistence. Rather than serving solely as a recovery phase, the upstroke appears to contribute to circulation buildup and transient force modulation, especially in hovering and low-speed ascent. The elevated Strouhal number observed during the thermal fling segment further supports the interpretation that butterflies temporarily prioritize unsteady force augmentation over steady propulsive efficiency.

These results reinforce the concept that flexible membrane wings can capture and redirect fluid volumes during stroke reversal, enhancing lift and thrust through controlled entrainment. The upstroke, therefore, must be incorporated explicitly into aerodynamic models of lepidopteran flight rather than treated as a purely drag-dominated phase.

### 4.3. Energy-Saving Flight Strategies and Ambient Flow Exploitation

The buoyancy-assisted ascent and plume riding sequences reveal a complementary strategy: butterflies can exploit weak ambient convection to reduce mechanical effort. Under thermal riding conditions, the wing motion shifts toward slower, large-amplitude deformations with reduced net translational demand. The inability to define a robust Strouhal number for near-hover regimes, due to near-zero reference velocity, highlights that classical scaling parameters become ill-conditioned when buoyancy and unsteady entrainment dominate.

This suggests a hybrid propulsion paradigm in which aerodynamic flapping and environmental energy harvesting coexist. Rather than generating all lift mechanically, butterflies appear capable of coupling flexible membranes to naturally occurring thermal gradients, effectively augmenting support through passive flow interaction. Such behaviour parallels observations in soaring birds but occurs at dramatically lower Reynolds numbers and spatial scales.

From an energetic standpoint, this strategy may reduce metabolic cost during low-speed ascent or station keeping. It also indicates that flow–structure interaction in butterflies extends beyond forced aerodynamic generation toward opportunistic environmental coupling.

### 4.4. Implications for Biomimetic Flapping Wing MAV Design

The findings have several implications for flapping wing micro air vehicles (FW-MAVs):

**Wing Cupping and Entrainment for Force Augmentation**

The visualization of cupped air release during clap–fling events suggests that controlled membrane curvature could be used to temporarily trap and redirect fluid volumes. Incorporating compliant membranes with programmable camber may enhance lift and thrust during stroke reversal in MAVs operating at low Reynolds numbers.

**Body Motion as a Control Input**

The pronounced thorax-driven manoeuvres indicate that distributed mass reconfiguration can serve as an effective control strategy. Rather than relying exclusively on wing kinematic asymmetry, future MAV architectures could integrate movable internal masses or compliant fuselage segments to emulate inertia redistribution observed in butterflies.

**Passive–Active Hybrid Strategies**

The ability to exploit ambient convection highlights the value of designing MAVs capable of interacting with environmental disturbances rather than resisting them. Flexible wings acting as low-power flow sensors and couplers could enable reduced actuation effort during plume-assisted ascent or hover.

**Aeroelastic Mode Matching**

The finite element model analysis indicates that the printed bio-inspired wings exhibit bending modes analogous to classical plate eigenmodes. Matching structural natural frequencies to wingbeat frequency ranges may allow beneficial passive deformation, amplifying aerodynamic efficiency through resonance-tuned compliance.

### 4.5. Relation to Previous Work and Novel Contributions

Previous studies have documented leading edge vortices, wake capture, and clap–fling aerodynamics primarily in dipteran and odonatan species [6,14]. Butterfly flight, characterized by lower frequency and higher membrane flexibility, has remained comparatively underexplored. The present work contributes by achieving the following:

• Providing high-speed schlieren visualization of freely flying butterflies.
• Demonstrating attached flow on cupped wings during the upstroke.
• Linking thorax-driven body reorientation directly to wake asymmetry.
• Quantifying Strouhal behaviour across ascent and plume-assisted regimes.

The seedless schlieren methodology avoids flow perturbation associated with PIV or smoke, enabling natural flight behaviour while still resolving coherent refractive index gradients. Although schlieren does not directly yield quantitative velocity fields, the combined kinematic reconstruction and phase-resolved visualization offer a robust qualitative–quantitative framework for interpreting unsteady mechanisms.

To improve readability and facilitate comparison between the analysed flight regimes, the principal kinematic parameters extracted from the schlieren recordings are summarized in Table 4. The table consolidates the measured translational velocities, wingbeat frequencies, and computed Strouhal numbers for the different flight conditions discussed in Section 3. This compact presentation highlights the variation in unsteady aerodynamic behaviour across ascent, plume-assisted, and manoeuvring cases.

### 4.6. Limitations and Future Directions

Several limitations must be acknowledged. Schlieren imaging captures density gradient structures rather than full velocity vectors, and interpretation of vortex cores relies on refractive index gradients. Additionally, specimen number and flight duration were constrained by laboratory conditions. Future work could integrate stereoscopic schlieren or background-oriented schlieren (BOS) for three-dimensional reconstruction, as well as synchronized force estimation through wake impulse methods.

Further research should explore the following:

• Coupled CFD–FSI simulations informed by experimentally extracted membrane kinematics.
• Closed-loop control strategies inspired by thorax-driven manoeuvring.
• Experimental validation of hybrid rotor flap configuration interacting with flexible membranes.

Future work will integrate velocity-resolved measurement techniques to enable direct application of the wake deficit formulation introduced in Section 2.3 and allow quantitative thrust and drag reconstruction.

Beyond the present geometry-driven prototype, future work will also incorporate advanced structural optimization methodologies to refine wing rigidity and compliance distribution. Generative design and topology optimization approaches can be employed to systematically tune venation thickness, membrane grading, and stiffness anisotropy under coupled aerodynamic–structural constraints. By integrating modal targets, flapping frequency ranges, and aeroelastic performance metrics into a multi-objective optimization framework, the wing structure can be mathematically tailored to promote beneficial passive deformation and resonance-tuned compliance. Such strategies have demonstrated effectiveness in other bio-inspired additive-manufactured systems and represent a promising direction for improving the aeroelastic efficiency of future flapping wing prototypes.

## 5. Conclusions and Future Work

This study presented high-speed schlieren visualization and kinematic analysis of free-flight butterfly aerodynamics, with particular emphasis on clap–fling interactions, membrane deformation, thorax-driven manoeuvring, and buoyancy-assisted ascent. The results demonstrate that butterfly flight at low Reynolds numbers is governed by strong aero–inertial coupling between flexible wings, body motion, and wake dynamics.

Several key conclusions can be drawn. First, schlieren imaging proved effective as a fully non-intrusive method for resolving unsteady flow structures in freely flying butterflies. The technique enabled direct visualization of entrained air volumes during clap–fling events, coherent wingtip vortex formation, and attached flow on cupped wings during the upstroke. These observations confirm that the upstroke contributes actively to force modulation rather than serving as a purely passive recovery phase.

Second, the experimental evidence highlights the dominant role of thorax and abdomen motion in rapid manoeuvring and attitude stabilization. The observed inversion–righting sequences and aggressive directional changes indicate that inertia redistribution and body axis reorientation are integral components of force generation and control. Butterfly flight therefore represents a distributed control system in which aerodynamic and inertial mechanisms are tightly coupled.

Third, the Strouhal number analysis across vertical ascent and plume-assisted regimes suggests that butterflies operate within or near biologically efficient flapping ranges during steady ascent, while temporarily shifting toward higher unsteady regimes during clap–fling-dominated phases. The plume-riding observations further indicate that butterflies can exploit ambient convection to reduce mechanical effort, supporting a hybrid passive–active propulsion strategy. For reference, efficient cruising flight in animals and bio-inspired flapping systems is commonly associated with Strouhal numbers in the range of 0.2–0.4. The measured value for vertical ascent (St ≈ 0.18) lies near the lower bound of this interval, indicating a balanced relationship between stroke amplitude and translational speed under controlled ascent conditions. In contrast, the higher Strouhal number observed during thermal riding (St ≈ 0.56) corresponds to a regime dominated by unsteady clap–fling interactions and reduced forward velocity, emphasizing transient force augmentation rather than steady propulsive efficiency.

From an engineering perspective, these findings directly inform the development of a bio-inspired flapping wing aerial scout prototype. The ultimate objective of this research is the construction of an Earth flight demonstrator that can later be adapted to Martian atmospheric conditions. The prototype is currently in its initial development phase and is conceived as a lightweight aerial reconnaissance platform, with low-speed manoeuvring, vertical ascent, and disturbance-tolerant flight defined as projected design objectives to be validated in future experimental stages.

Future development of the prototype will focus on controlled membrane cupping and aeroelastic mode tuning. Membrane compliance will be adjusted through graded thickness and venation anisotropy to promote passive camber formation during clap–fling interaction. In parallel, parametric finite element optimization will be employed to align bending mode frequencies with the operational wingbeat range, enabling resonance-tuned aeroelastic coupling. Additive manufacturing allows systematic variation of venation geometry and stiffness distribution to achieve these objectives.

For Earth-based operation, the prototype will focus on the following:

• Flexible membrane wings with controlled cupping for clap–fling force augmentation.
• Structural mode tuning through additive manufacturing to achieve favourable aeroelastic coupling.
• Integration of body segment or internal mass motion to emulate thorax-driven manoeuvring.
• Hybrid flight strategies capable of interacting with ambient flow disturbances rather than resisting them.

For Martian adaptation, additional considerations include the following:

• Operation in a low-density CO_2_ atmosphere with significantly reduced Reynolds numbers.
• Increased stroke amplitude and frequency requirements to compensate for reduced aerodynamic force.
• Structural mass optimization and material selection for extreme temperature ranges.
• Potential hybridization with auxiliary propulsion (e.g., rotor-assisted or impulsive boost mechanisms) to ensure takeoff robustness.

Future work will therefore proceed along four main directions, as follows:

• Construction and experimental validation of the first Earth flight prototype using the additively manufactured wing structures characterized in this study.
• Coupled CFD–FSI simulations incorporating experimentally derived membrane kinematics to refine aerodynamic performance predictions.
• Development of bio-inspired control strategies based on thorax-driven inertia redistribution and distributed aeroelastic feedback.
• Scaling analysis and atmospheric adaptation studies to assess feasibility under Martian gravity and atmospheric density conditions.

In summary, this work establishes a foundational experimental and analytical framework linking butterfly unsteady aerodynamics to the design of a next-generation bio-inspired aerial scout. By combining clap–fling aerodynamics, membrane flexibility, and body-driven control, the proposed prototype aims to bridge biological inspiration and aerospace engineering, progressing from Earth-based validation toward future planetary exploration applications.

## Figures and Tables

**Table 1:** Approximate Reynolds number ranges associated with the flight of selected insect groups.

Insect Type	Approx. Re Number
Thrips (tiny insects)	~10 [10,11]
Fruit fly (Drosophila sp.)	~120 [1]
Vegetable leafminer/small dipterans	~21–40 [12]
Honeybee/Bumblebee (Apis, Bombus)	~230–3000 (varies by species/flight condition) [13]
Hawkmoth/larger Lepidoptera	~5000–6000 [1]
Large dragonflies (Odonata)	~1000 [14]
General insect flight range	~10–10^4^ [1,2]

**Table 2:** Schlieren equipment data and description.

Component	Model Specifications	Key Parameters	Notes
Parabolic mirrors	Al-plated	EFL = 1524 mm	Used in Toepler—single mirror
	From Edmund Optics [32]	Diameter = 254 mm λ/8	Used in Z-type/shadowgraph
Light source	LS-S1 [33]	Output diameter 0.5 mm	Laser-pumped, wavelength 440–750 nm
High-speed camera	Phantom Veo 10 L [34]	Max. resolution 120 × 820 at max. 7500 fps	Used at different recording speed
Camera lens	Tamron Nikon [35]	70–200 mm F/2.8, MACRO	Used with adjusted zoom and diaphragm opening
Knife edge	Anti-reflective black metal sheet	-	Mounted on tripod
Data acquisition system	Alienware M15	Ethernet cable connection,	Live feed, downloading videos from camera buffer

**Table 3:** Camera settings and recording purpose.

Video	Settings	Analysed Specimen	Purpose of Recording
1	Camera: Resolution: 1280 × 800, 7500 fps, exposure: 0.5 μs Z-type schlieren	Iphiclides podalirius	Control mechanism by thorax swing and wing deformation
			Trajectory reconstruction
2	Resolution: 1280 × 800, 7500 fps, exposure: 1 μs	Pieris rapae	Thermal fling
3			Normal upstroke–downstroke visualization
4			Extreme manoeuvring case
5			Vertical plane walk
6			Wing deformation
7			Tip wing vortex tracking

**Table 4:** Flight kinematics summary.

Flight Condition	Mean Velocity [m/s]	Wingbeat Frequency [Hz]	Strouhal Number
Diagonal flight	1.675	~15–16	-
Vertical ascent	1.236	13.3	0.18
Thermal riding	Low (plume-assisted)	~13–15 *	0.56
Near hover	0	~13–15 *	Not reported

* Frequency range inferred from described wingbeat behaviour (≈15–16 Hz mentioned in manoeuvring case).

## Data Availability

All generated data are contained in the article or can be made available upon request.
